# Körperliche Aktivität bei Patienten mit Kopf-Hals-Tumoren: Analyse subjektiver Einstellungen, Symptomlast und Akzelerometriedaten

**DOI:** 10.1007/s00106-026-01768-8

**Published:** 2026-05-04

**Authors:** Sabine Felser, Christina Grosse-Thie, Julia Daunheimer, Daniel Strüder, Chia Jung Busch, Christian Junghanss, Sabina Ulbricht

**Affiliations:** 1 Klinik für Hämatologie, Hämostaseologie, Onkologie, Stammzelltherapie und Palliativmedizin, Department für Innere Medizin, Universitätsmedizin Rostock, Ernst-Heydemann-Str. 06, 18057 Rostock, Deutschland; 2 Hämatologische und Onkologische Praxis, Rostock, Deutschland; 3 Klinik und Poliklinik für Hals-Nasen-Ohrenheilkunde, Kopf- und Halschirurgie „Otto Körner“, Universitätsmedizin Rostock, Rostock, Deutschland; 4 Klinik und Poliklinik für Hals‑, Nasen‑, Ohrenkrankheiten, Universitätsmedizin Greifswald, Greifswald, Deutschland; 5 Institut für Community Medicine, Abteilung SHIP-KEF, Universitätsmedizin Greifswald, Greifswald, Deutschland

**Keywords:** Bewegungsmangel, Gesundheitsbezogene Lebensqualität, Fatigue, Lebensstilfaktoren, Akzelerometrie, Sedentary behavior, Health-related quality of life, Fatigue, Lifestyle factors, Accelerometry

## Abstract

**Hintergrund:**

Körperliche Aktivität unterstützt die physische und psychosoziale Rehabilitation nach Krebs. Die Aktivierung von Patienten mit Kopf-Hals-Tumoren gelingt bislang nur schwer. In der Studie wurde der Zusammenhang zwischen der subjektiv eingeschätzten Wichtigkeit, körperlich aktiver zu werden, und dem aktuellen Aktivitätsniveau sowie soziodemografischen und klinischen Faktoren untersucht.

**Methoden:**

In einer prospektiven Querschnittstudie wurden ambulante Patienten mit Kopf-Hals-Tumoren (≥ 18 Jahre) eingeschlossen. Erfasst wurden soziodemografische Daten, die Symptomschwere (u. a. Fatigue, Schmerz) mittels visueller Analogskalen (VAS, 0–10), Dyspnoe (mMRC-Skala [Modified British Medical Research Council]) sowie die Wichtigkeit der Aktivitätssteigerung (VAS 0–10). Die körperliche Aktivität wurde per 7‑tägiger Akzelerometrie erfasst (inkludiert: ≥ 10 h/Tag an ≥ 4 Tagen). Die Auswertung erfolgte uni-, bi- und multivariat.

**Ergebnisse:**

Insgesamt wurden 51 Patienten (82 % Männer, Durchschnittsalter 67 ± 19 Jahre) eingeschlossen; 39 davon lieferten valide Akzelerometriedaten. Eine Aktivitätssteigerung wurde von 68 % der Teilnehmer als wichtig bzw. sehr wichtig bewertet. Im Mittel waren die Teilnehmer 179 ± 89 min/Tag körperlich aktiv. Bei Alleinlebenden (*p* = 0,033) und Teilnehmern mit erhöhter Dyspnoe (*p* = 0,056) oder stärkeren Schmerzen (*p* = 0,068) war die Wichtigkeit, sich mehr zu bewegen, ausgeprägter.

**Schlussfolgerung:**

Patienten mit Kopf-Hals-Tumoren haben ein niedriges Aktivitätsniveau mit durchschnittlich 3 h Bewegung am Tag. Für die Mehrheit der Teilnehmer ist es wichtig, sich mehr zu bewegen. Welche Rolle eine höhere Symptomlast und soziale Faktoren dabei spielen, der Bedeutung von Bewegung mehr Gewicht beizumessen, sollte Gegenstand weiterer Forschung sein.

## Abstract (en)

**Background:**

Physical activity (PA) supports physical and psychosocial rehabilitation after cancer. However, motivating patients with head and neck cancer (HNC) to become more active remains challenging. This study investigated the association of the self-rated importance of increasing PA with current activity levels as well as sociodemographic and clinical factors.

**Methods:**

In this prospective cross-sectional study, outpatient HNC patients (≥ 18 years) were included. Sociodemographic data, symptom burden (e.g., fatigue, pain) using visual analog scales (VAS; 0–10), dyspnea (modified Medical Research Council scale, mMRC), and the perceived importance of becoming more active (VAS 0–10) were recorded. Current PA was assessed via 7‑day accelerometry (valid if ≥ 10 h/day on ≥ 4 days). Analyses were conducted using univariate, bivariate, and multivariate methods.

**Results:**

A total of 51 patients (82% male; mean age 67 ± 19 years) were included; 39 provided valid accelerometry data. Increasing PA was rated as important or very important by 68% of participants. On average, participants were physically active for 179 ± 89 min per day. The perceived importance of becoming more active was higher among those living alone (*p* = 0.033) and tended to be higher in participants with greater dyspnea (*p* = 0.056) or more severe pain (*p* = 0.068).

**Conclusion:**

The average activity level in this sample was low, with around 3 h of movement per day. For the majority of participants, increasing activity was important. Further research is needed to clarify why higher symptom burden and social factors are associated with a greater perceived importance of becoming more active.

Kopf-Hals-Tumoren gehören weltweit zu den häufigsten Krebserkrankungen mit hoher Mortalität und erheblicher Symptomlast. Körperliche Aktivität kann die Lebensqualität Betroffener deutlich verbessern, wird jedoch selten umgesetzt. Besonders in strukturschwachen Regionen und im ländlichen Raum fehlt es an niederschwelligen Bewegungsangeboten. Um möglichst individualisierte Empfehlungen aussprechen zu können, müssen die Perspektiven der Patienten besser verstanden werden.

Kopf-Hals-Tumoren („head and neck cancers“, HNC) sind weltweit für über 685.000 Fälle und 375.000 Todesfälle pro Jahr verantwortlich und gehören damit zu den sechs häufigsten Krebserkrankungen [31]. In Deutschland werden jährlich bei etwa 10.000 Männern und 4500 Frauen HNC diagnostiziert [27]. Aufgrund sozioökonomischer Benachteiligung und der hohen Prävalenz veränderbarer Risikofaktoren wie Rauchen und Alkoholkonsum sind die Inzidenz und Mortalität im Zusammenhang mit HNC in Mecklenburg-Vorpommern (M-V) am höchsten [28].

Die Mehrheit der Patienten mit HNC leidet aufgrund der Lage der Tumoren und der intensiven lokalen und systemischen Therapieverfahren unter einer hohen und zum Teil chronischen Symptombelastung, darunter Dysphonie, Dysphagie, Gewichtsverlust, Sarkopenie, Fatigue, eingeschränkte Mobilität und psychosoziale Probleme. Diese hohe Symptomlast beeinträchtigt die gesundheitsbezogene Lebensqualität der Betroffenen [4, 7, 18].

Der Nutzen von körperlicher Aktivität für die Muskelkraft, kardiorespiratorische Fitness, die Mobilität, die Schmerzlinderung und die Verbesserung der Lebensqualität HNC-Patienten ist gut dokumentiert [1, 6, 26]. Daten aus Taiwan und Schweden zeigen, dass HNC-Patienten körperlich unzureichend aktiv sind [10, 16].

Um HNC-Patienten, welche keinen Zugang zu strukturierten Bewegungsprogrammen haben (z. B. in ländlichen Regionen) oder ein Training zu Hause bevorzugen, möglichst individualisierte Bewegungsempfehlungen geben zu können, wurden im Rahmen der multizentrischen OSHO-#94-Studie die Machbarkeit, Sicherheit und Effekte eines individualisierten Heimtrainings evaluiert [12]. Die Rekrutierung erwies sich hierbei als herausfordernd. Daten einer Zwischenanalyse zur Rekrutierung an der Klinik und Poliklinik für Hals-Nasen-Ohrenheilkunde, Kopf- und Halschirurgie „Otto Körner“ in Rostock zufolge lag die Rekrutierungsquote bei nur 7 % (9 von 134 angesprochenen Patienten nahmen teil) [14]. Mit Unterstützung des deutschlandweiten Selbsthilfenetzwerk M.U.N.D. gelang der Einschluss von 53 Patienten in die Studie. Entsprechend zeigte sich der Selektions-Bias daran, dass die Stichprobe einen Anteil von 43 % Frauen umfasste, 38 % der Teilnehmenden Abitur hatten, 58 % Nichtraucher waren und 60 % angaben, vor Erkrankung sportlich aktiv gewesen zu sein [11]. Folglich spiegelt die Stichprobe in der OSHO-#94-Studie den typischen HNC-Patienten nur sehr eingeschränkt wider. Dieser ist männlich, hat ein geringes Bildungsniveau und raucht [2].

Um die Reichweite und Repräsentativität der Zielgruppe in Bewegungsprogrammen zu verbessern, haben wir eine Studie konzipiert, um (1.) den Stellenwert zu untersuchen, den HNC-Patienten einer Steigerung ihres Aktivitätsniveaus beimessen, und (2.) ob dieser mit ihrer aktuellen Aktivität, erfasst mittels Akzelerometer, sowie (3.) soziodemografischen und klinischen Faktoren zusammenhängt.

## Methodik

### Studiendesign und Einschlusskriterien

Es handelt sich um eine prospektive Querschnittstudie. Ziel war es, ≥ 35 Teilnehmer zu rekrutieren. Einschlusskriterien waren: Diagnose nach ICD C00–C14, C30–C32 (HNC), Alter ≥ 18 Jahre, in ambulanter Therapie oder Nachsorge, ausreichende Deutschkenntnisse und Gehfähigkeit. Die Teilnahme an der Studie war nur nach schriftlicher Zustimmung möglich. Rekrutiert wurden die Teilnehmer zwischen Januar und September 2022 während der Sprechstunde in der Universitätsmedizin Rostock und in der Rostocker Selbsthilfegruppe für HNC.

### Fragebögen

Nach Studieneinschluss füllten die Teilnehmer einen Fragebogen aus. Dieser umfasste:

*Patientencharakteristika: *Erfragt wurden Geschlecht, Alter, Größe, Gewicht, Schulbildung, subjektiver Sozialstatus (SSS), Lebensumfeld (städtisch vs. ländlich), Lebensgemeinschaft (ja vs. nein), sportliche Betätigung vor der HNC-Diagnose, Rauchen (ja vs. nein) und Alkoholkonsum (ja vs. nein). Der Body-Mass-Index (BMI) wurde berechnet (Körpergewicht/Körpergröße zum Quadrat, kg/m^2^) und in BMI < 18,5 (= Untergewicht); 18,5 bis 24,9 (= Normalgewicht); ≥ 25 bis 29,9 (= Übergewicht) und ≥ 30 (= Adipositas) eingeteilt. Basierend auf dem deutschen Schulsystem wurden die Schuljahre in < 10, = 10 oder > 10 Jahre eingeteilt. Der SSS wurde anhand der MacArthur-Skala erhoben [25]. Präsentiert wurden eine 10-stufige Leiter und die Frage: „Stellen Sie sich diese Leiter als Symbol für die Stellung der Menschen in unserer Gesellschaft vor, einschließlich mehr Geld, mehr Bildung und bessere Arbeitsplätze. Höhere Sprossen stehen für einen höheren sozialen Status.“ Die Antworten wurden wie folgt kategorisiert: niedriger SSS (Sprossen 1–3), mittlerer SSS (Sprossen 4–7) und höherer SSS (Sprossen 8–10).

*Symptomlast:* Erfasst wurden die bewegungstherapierelevanten Symptome Schlafqualität, Fatigue, Schmerzen und Dyspnoe. Die Schlafqualität wurde anhand einer visuellen Analogskala (VAS) von 0 (sehr schlecht) bis 10 (sehr gut) bewertet. Fatigue und Schmerzen wurden anhand einer VAS von 0 (gar nicht/keine) bis 10 (sehr häufig/sehr stark) bewertet. Dyspnoe wurde anhand der modifizierten Dyspnoe-Skala des Medical Research Council [20] erfasst und in „ja“ oder „nein“ dichotomisiert.

*Wichtigkeit, körperlich aktiver zu werden: *Die Teilnehmer bewerteten die Wichtigkeit, ihr Aktivitätsniveau zu steigern, auf einer VAS von 0 (nicht wichtig) bis 10 (sehr wichtig). Die Antworten wurden in zwei Gruppen unterteilt: nicht/weniger wichtig (0 bis 3) und wichtig/sehr wichtig (≥ 4).

Aus den Arztberichten wurden folgende *klinische Daten* erfasst: Monat und Jahr der Erstdiagnose, Tumorlokalisation, UICC-Stadium, aktuelle Therapiesituation, vollständige Remission und erhaltene Therapien.

### Akzelerometereinsatz

Das für den Tragezeitraum initialisierte Gerät wurde an die Teilnehmer ausgehändigt. Sie wurden gebeten, das Akzelerometer sieben Tage lang möglichst durchgängig an einem flexiblen Gürtel an der Taille zu tragen. Das Gerät sollte nur nachts und bei Aktivitäten mit Wasserkontakt (z. B. Schwimmen und Duschen) abgelegt werden. Nach Ablauf der Tragezeit wurde das Akzelerometer an die Studienzentrale zurückgeschickt. Daraufhin erhielten die Teilnehmer per Post die zu Beginn angekündigte Aufwandsentschädigung in Höhe von 25 €.

Die Daten wurden mit einem dreiachsigen ActiGraph-Beschleunigungsmesser Modell GT3X+ (Fa. ActiGraph, Pensacola/FL, USA) mit einer Abtastrate von 30 Hz erfasst. Für die Auswertung wurden nur Daten der vertikalen Achse verwendet. Nach dem Auslesen der Daten wurden diese mit der ActiLife-Software (Version 6.13.3; ActiGraph) verarbeitet.

Aus den vorprozessierten, auf Epochen von 10 s bezogenen gerätespezifischen „counts per minute“ (cpm) wurden nach Ausschluss von Nichttragezeiten die interessierenden Endpunkte mithilfe von „cutpoints“ berechnet. Als Nichttragezeit des Akzelerometers wurde definiert, dass an mindestens 60 aufeinanderfolgenden Minuten keine Aktivitätsintensität aufgezeichnet wurde, wobei ≤ 2 min zwischen 0 und 100 Zählungen (cpm) zulässig waren. Unter Nutzung des Trojano-Algorithmus für Erwachsene [32] wurden die Zeiten mit sitzender Tätigkeit (< 100 cpm), leichter körperlicher Aktivität (100 bis 2019 cpm), moderater körperlicher Aktivität (2020 bis 5998 cpm) und intensiver körperlicher Aktivität (> 5999 cpm) in Minuten pro Tag (min/Tag) ermittelt. In die Analyse gingen nur Teilnehmer ein, die das Gerät an mindestens 4 Tagen (einer davon ein Wochenendtag) und pro Tag mindestens 10 h getragen hatten [23].

### Statistische Analyse

Kontinuierliche Daten werden als Mittelwert ± Standardabweichung einschließlich Median, Minimum und Maximum angegeben. Kategoriale Daten werden als Anzahl (*n*) und Prozent dargestellt. Kontinuierliche Daten wurden mit dem Shapiro-Wilk-Test auf Normalverteilung geprüft. Zusammenhänge wurden mittels Pearson- oder Spearman-Korrelation geprüft. Zur Interpretation der Zusammenhänge wurde der Korrelationskoeffizient r verwendet. Gruppenunterschiede wurden mit dem Mann-Whitney-U-Test (asymptotisch) geprüft; zum Vergleich von Anteilen wurde der χ^2^-Test verwendet.

Für die Gesamtstichprobe (*N* = 51) wurde eine multivariable logistische Regression berechnet. Faktoren, die in bivariaten Vergleichen einen Wert von *p* < 0,10 aufwiesen, wurden in der Regressionsmodellierung berücksichtigt, wobei „*Wichtigkeit, körperlich aktiver zu werden*“ als Ergebnismaß verwendet wurde. Die vier Ausprägungen dieser als abhängig verwendeten Variable wurden dichotomisiert (Kategorie 1: „nicht/weniger wichtig“, Kategorie 2: „wichtig/sehr wichtig“). Das logistische Regressionsmodell wurde um das Alter und die Zeit nach der Erstdiagnose bereinigt.

Alle Daten wurden mit SPSS (Version 25.0, Fa. IBM, Chicago/IL, USA) analysiert. Die statistische Signifikanz wurde bei *p*-Werten < 0,05 angenommen. Aufgrund der geringen Stichprobengröße wurden schwache Belege gegen die Nullhypothese für die multivariable logistische Regression bei *p*-Werten zwischen ≥ 0,05 und < 0,1 akzeptiert und explorativ interpretiert [17].

## Ergebnisse

### Patientencharakteristika

**Abb. 1:**
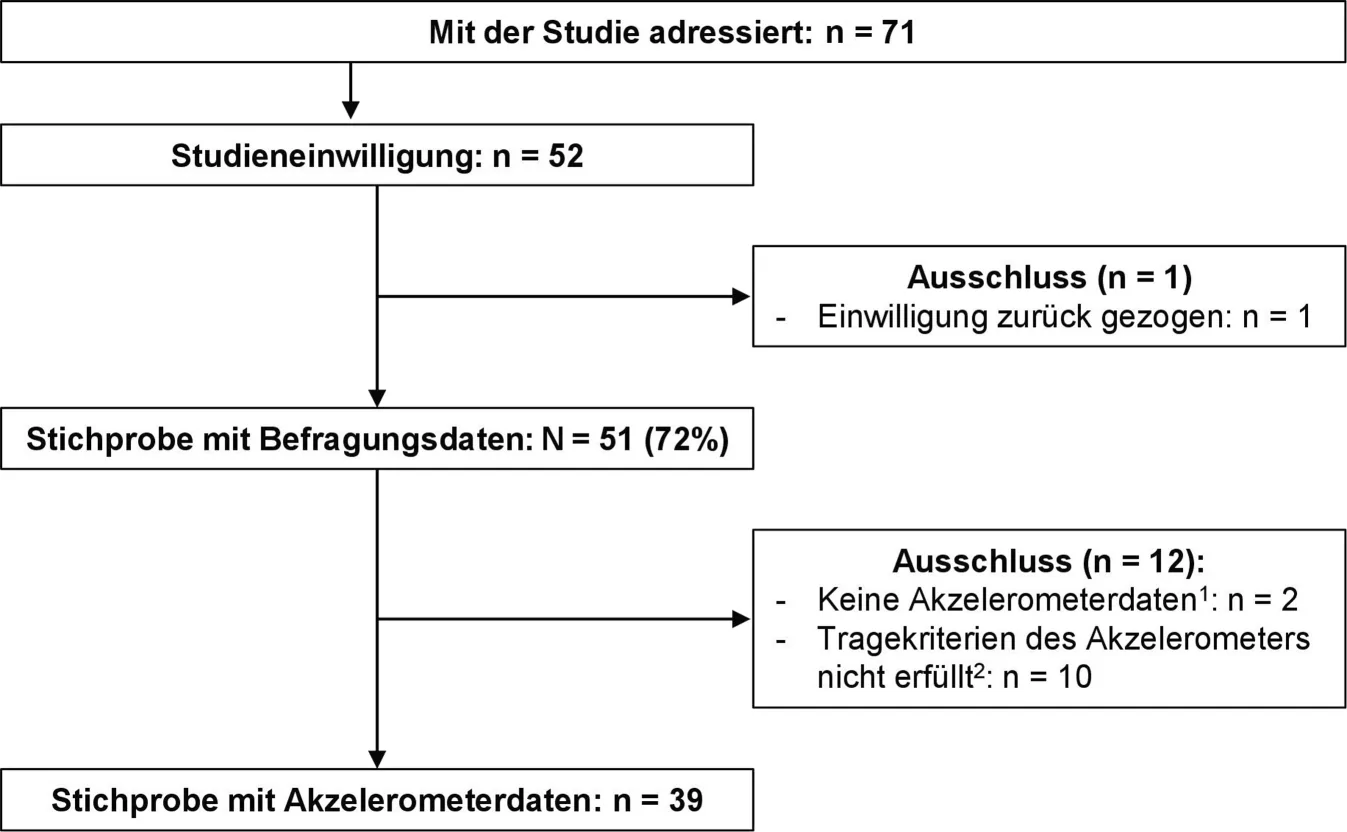
Flussdiagramm. Rekrutierungszeitraum: Januar bis September 2022. ^1^ Technische Fehler während der Initialisierung der Geräte. ^2^ Akzelerometer wurde an mindestens 4 von 7 Tagen ≥ 10 h/Tag getragen, darunter mindestens ein Wochenendtag

Insgesamt wurde 71 HNC-Patienten die Studienteilnahme angeboten, 52 von ihnen gaben ihre Einwilligung. Da ein Patient seine Einwilligung zurückzog, nahmen 51 Patienten (71,8 %) an der Studie teil (Abb. 1). Die Mehrheit von ihnen war männlich (82 %, *n* = 42), das Durchschnittsalter lag bei 67 ± 10 Jahren, und 82 % (*n* = 42) hatten maximal die 10. Klasse abgeschlossen (Tab. 1). Etwa die Hälfte der Teilnehmer (47 %, *n* = 24) lebte in einer ländlichen Region, 41 % (*n* = 21) waren alleinlebend. Der Anteil der Raucher lag bei 31 % (*n* = 16, durchschnittlich 13 ± 9 Zigaretten pro Tag) und weitere 57 % (*n* = 29) gaben an, Exraucher zu sein. Fast die Hälfte (47 %, *n* = 24) gab an, keinen Alkohol zu konsumieren.

**Tab. 1:** Soziodemografische und klinische Daten der Stichprobe (*n* = 51)

Variable	Kategorie	*n* (%) bzw. MW ± SD (Min–Max)
Geschlecht	Weiblich	9 (18)
	Männlich	42 (82)
Alter (Jahre)	–	66,8 ± 9,5 (38–86)
Body Mass Index (kg/m^2^)	–	25,7 ± 5,1 (16,3–37,2)
	< 18,5	5 (10)
	18,5–24,9	19 (37)
	25,0–29,9	18 (35)
	≥ 30,0	9 (18)
Schulbildung (Jahre)	< 10	14 (27)
	10	28 (55)
	> 10	9 (18)
Subjektiver Sozialstatus (10-sprossige Leiter)	–	5,7 ± 1,6 (2–9)
	Niedrig (Sprossen ≤ 3)	6 (12)
	Mittel (Sprossen 4 bis 7)	39 (76)
	Hoch (Sprossen ≥ 8)	6 (12)
Lebensumfeld	Städtisch	27 (53)
	Ländlich	24 (47)
Lebensgemeinschaft	Ja	30 (59)
	Nein	21 (41)
Sportlich aktiv vor Diagnose	Ja	20 (39)
	Nein	31 (61)
Tabakkonsum	Ja	16 (31)
	Nein	35 (69)
Alkoholkonsum	Ja	27 (53)
	Nein	24 (47)
Zeit nach Diagnose (Monate)	–	57 ± 53 (5–271)
Tumorlokalisation	Oropharynx	21 (41)
	Larynx	10 (20)
	Hypopharynx	6 (12)
	Sonstige	14 (27)
UICC-Stadium	I/II	14 (27)
	III/IV	37 (73)
Aktuelle Therapiephase	Nachsorge	34 (67)
	Aktive Therapie	17 (33)
Therapien^a^	Nur Op.	7 (14)
	Nur RT oder RCT	17 (34)
	Op. und RT/RCT	26 (52)

*n* Anzahl der Patienten, *MW* Mittelwert, *SD* „standard deviation“, Standardabweichung, *Min* Minimum, *Max* Maximum, *Op.* Operation, *RT* Radiotherapie, *RCT* kombinierte Radiochemotherapie

^a^ Zehn Patienten sind unter palliativer Checkpointinhibitortherapie

Die Erstdiagnose HNC erhielten die Teilnehmer zwischen Januar 2000 und November 2021. Die häufigste Tumorlokalisation war der Oropharynx (41 %, *n* = 21). Zum Zeitpunkt der Studie erhielten 20 % (*n* = 10) der Teilnehmer eine Checkpointinhibitortherapie mit palliativer Intention. Aufgrund der Tatsache, dass sich ein Teilnehmer unter Checkpointinhibition in vollständiger Remission befand, waren 69 % (*n* = 35) der Teilnehmer in vollständiger Remission.

### Symptomlast

Der Durchschnittswert für die Schlafqualität lag bei 6,4 ± 3,0. Ein Anteil von 29 % (*n* = 15) gab an, keine Fatigue zu verspüren, während 25 % (*n* = 13) und 45 % (*n* = 23) angaben, dieses Symptom selten bzw. häufiger zu haben. Fast die Hälfte der Teilnehmer gab an, schmerzfrei zu sein (47 %, *n* = 24). Leichte, mäßige oder starke Schmerzen wurden von 35 % (*n* = 12), 20 % (*n* = 10) bzw. 10 % (*n* = 5) der Teilnehmer angegeben. Während 41 % (*n* = 21) keine Dyspnoe angaben, berichteten 47 % (*n* = 24) von Atemnot beim schnellen Gehen oder bei leichter Steigung und 12 % (*n* = 6) von schwereren Problemen (Tab. 2).

**Tab. 2:** Symptomlast und akzelerometerbasierte körperliche Aktivität

Variable	*n* (%) bzw. MW ± SD (Min–Max)
**Symptomlast (*n*** **=** **51)**	
*Schlafqualität (0 „sehr schlecht“ bis 10 „sehr gut“)*	6,4 ± 3,0 (0–10)
*Fatigue (0 „gar nicht“ bis 10 „sehr oft“)*	3,7 ± 3,5 (0–10)
*Schmerz (0 „keine“ bis 10 „sehr starke“)*	2,3 ± 2,7 (0–9)
*Dyspnoe*	
Keine Beschwerden	21 (41)
Kurzatmig beim raschen Gehen/leichten Anstieg	24 (47)
Kurzatmig beim Gehen in der Ebene/Pausen zum Atemholen	1 (2)
Benötige Pausen beim Gehen nach einigen Minuten/kurzer Strecke	4 (8)
Zu kurzatmig, um das Haus zu verlassen/Luftnot beim Reden, Ankleiden	1 (2)
**Akzelerometerbasierte körperliche Aktivität (min/Tag) (*n*** **=** **39)**	
*Sitzende Aktivitäten*	615 ± 139 (357–1120)
*Leichte körperliche Aktivität*	160 ± 78 (38–422)
*Moderate körperliche Aktivität*	19 ± 20 (0–90)
*Intensive körperliche Aktivität*	0 ± 1 (0–3)
*Körperliche Aktivität gesamt*	179 ± 89 (38–435)

*n* Anzahl der Patienten, *MW* Mittelwert, *SD* Standardabweichung, *Min* Minimum, *Max* Maximum

### Akzelerometerbasierte körperliche Aktivität

Laut Akzelerometriedaten verbrachten die Teilnehmer durchschnittlich 615 ± 139 min/Tag mit sitzenden Aktivitäten. Aus den Daten ging keine Zeit mit intensiven körperlichen Aktivitäten hervor. Die durchschnittliche Zeit in moderater körperlicher Aktivität lag bei 19 ± 20 min/Tag (Median 12 min/Tag); die durchschnittliche Zeit in leichter Aktivität lag bei 160 ± 78 min/Tag (Median 154 min/Tag). Im Durchschnitt waren die Teilnehmer 179 ± 89 min/Tag körperlich aktiv. Der Median lag bei 169 min/Tag.

### Wichtigkeit, körperlich aktiver zu werden

**Abb. 2:**
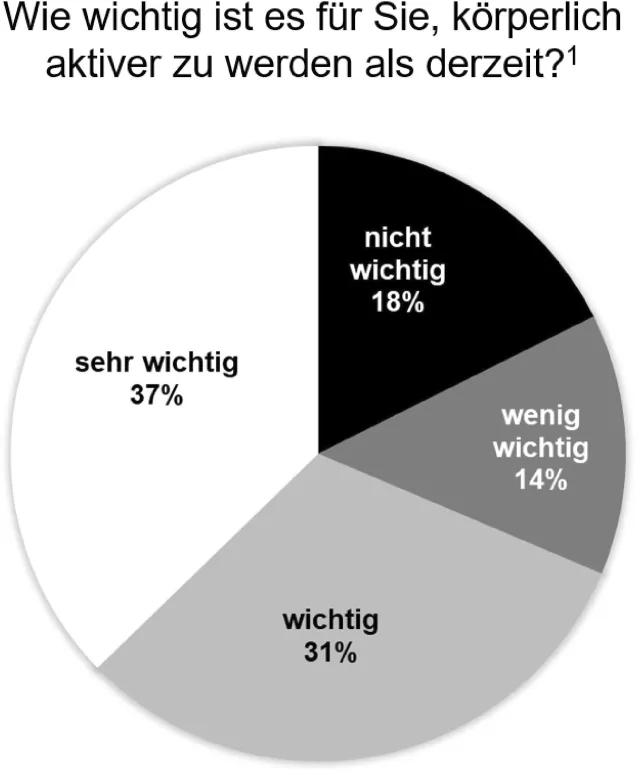
Einstellung von Patienten mit Kopf-Hals-Tumoren gegenüber körperlicher Aktivität (*n* = 51). ^1^ Visuelle Analogskala von 0 „überhaupt nicht wichtig“ bis 10 „sehr wichtig“. Kategorisierung: 0 „nicht wichtig“, 1–3 „wenig wichtig“, 4–6 „wichtig“, ≥ 7 „sehr wichtig“

**Abb. 3:**
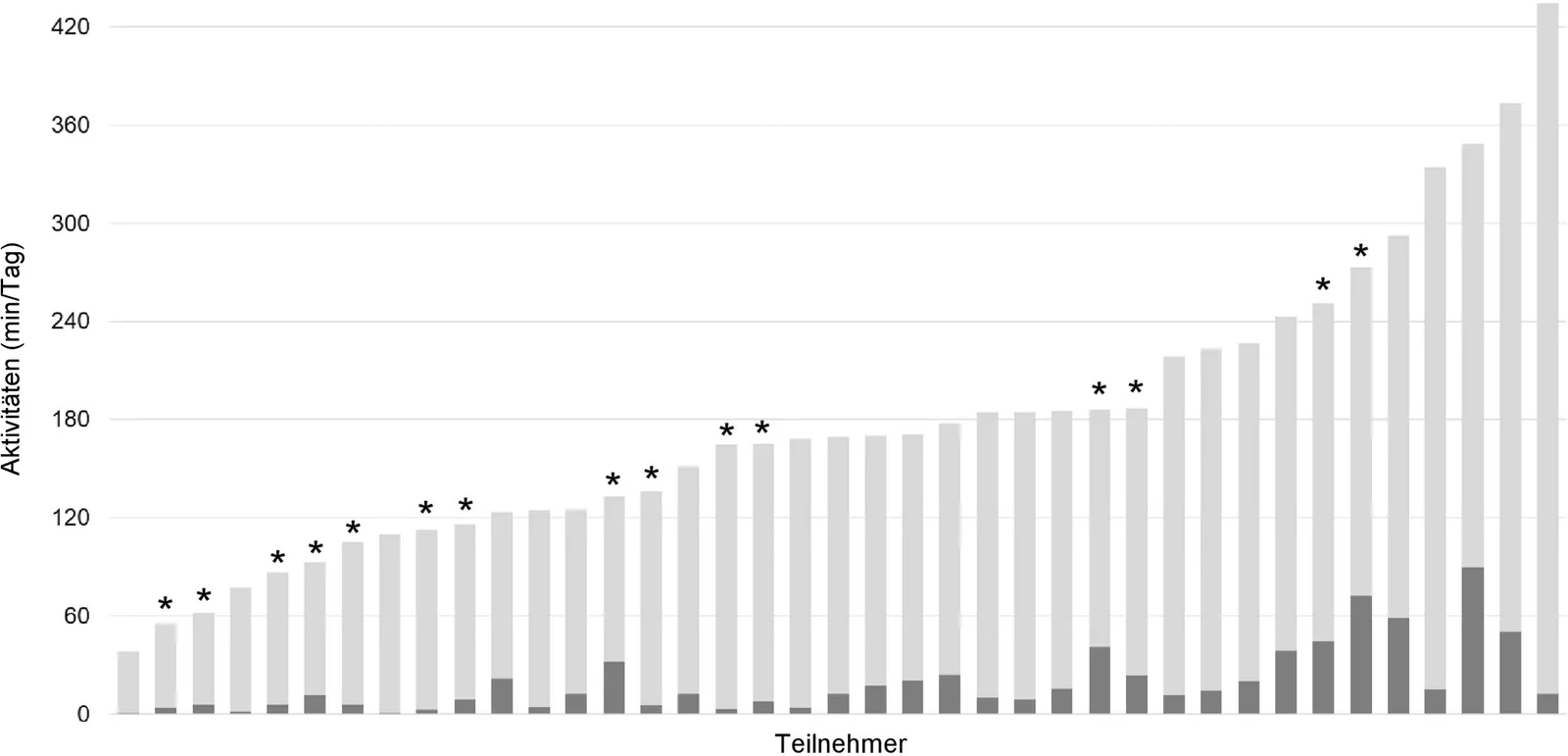
Umfang und Intensität der akzelerometerbasierten körperlichen Aktivität (*n* = 39). *Hellgrau* = leichte körperliche Aktivität; *dunkelgrau* = moderate körperliche Aktivität. * Teilnehmer, die angaben, dass eine Steigerung der körperlichen Aktivität sehr wichtig ist. Altersadjustierte Korrelation zwischen Gesamtaktivität und Wichtigkeit, körperlich aktiver zu werden: r = −0,365; *p* = 0,024

Insgesamt 68 % (*n* = 35) der Teilnehmer gaben an, dass es ihnen wichtig bzw. sehr wichtig sei, ihr Aktivitätsniveau zu steigern (Abb. 2). Es wurden moderate Korrelationen zwischen dem Umfang an leichter körperlicher Aktivität sowie der Gesamtaktivität und der Wichtigkeit, körperlich aktiver zu werden, festgestellt (r = −0,378; *p* = 0,018 bzw. r = −0,335; *p* = 0,037) (Abb. 3).

### Zusammenhänge zwischen körperlicher Aktivität und Symptomen

Die Korrelationsmatrix zeigte einen moderaten negativen Zusammenhang zwischen dem Umfang an moderater körperlicher Aktivität und dem Alter (r = −0,490, *p* = 0,002). Altersbereinigt zeigten sich keine signifikanten Korrelationen zwischen der gesamten körperlichen Aktivität und der Symptomlast.

### Prädiktoren für mehr körperliche Aktivität

Wir verglichen soziodemografische und klinische Daten der Teilnehmer und legten dabei den Schwerpunkt auf Vergleiche zwischen denjenigen, die angaben, dass es ihnen nicht oder weniger wichtig sei, körperlich aktiver zu werden, und denjenigen, die eine höhere Wichtigkeit angaben. Signifikante Unterschiede zwischen den beiden Gruppen wurden bei den Variablen „Lebensgemeinschaft“ (*p* = 0,035) und den Symptomen Fatigue (*p* = 0,015) und Schmerzen (*p* = 0,010) sichtbar (Tab. 3).

**Tab. 3:** Vergleich ausgewählter Variablen, klassifiziert nach der Wichtigkeit, körperlich aktiver zu werden (*n* = 51)

Variable	Wichtigkeit, körperlich aktiver zu werden:		*p*-Wert^1^
	Keine/gering	Hoch/sehr hoch	
	(*n* = 16)	(*n* = 35)	
**Geschlecht**	–	–	0,701
Weiblich	2 (22)	7 (78)	–
Männlich	14 (33)	28 (67)	–
**Alter (Jahre)**	66,0 ± 10,0	68,6 ± 8,4	0,382
**Body Mass Index (kg/m**^**2**^**)**	25,3 ± 4,6	26,5 ± 6,1	0,605
**Schulbildung (Jahre)**	–	–	0,777
< 10	4 (29)	10 (71)	–
10	10 (36)	18 (64)	–
> 10	2 (22)	7 (78)	–
**Subjektiver Sozialstatus (10-sprossige Leiter)**	–	–	0,533
Niedrig	1 (17)	5 (83)	–
Mittel	14 (36)	25 (64)	–
Hoch	1 (17)	5 (83)	–
**Lebensumfeld**	–	–	1,000
Städtisch	8 (30)	19 (70)	–
Ländlich	8 (33)	16 (67)	–
**Lebensgemeinschaft**	–	–	0,035*
Ja	13 (43)	17 (57)	–
Nein	3 (14)	18 (86)	–
**Sportlich aktiv vor Diagnose**	–	–	0,543
Ja	5 (25)	15 (75)	–
Nein	11 (36)	20 (64)	–
**Tabakkonsum**	–	–	0,329
Ja	3 (19)	13 (81)	–
Nein	13 (37)	22 (63)	–
**Alkoholkonsum**	–	–	1,000
Ja	8 (30)	19 (70)	–
Nein	8 (33)	16 (67)	–
**Zeit nach Diagnose (Monate)**	63 ± 64	54 ± 49	0,496
**Komplette Remission**	–	–	0,746
Ja	12 (34)	23 (66)	–
Nein	4 (25)	12 (75)	–
**Symptome**			
*Schlafqualität* (0 „sehr schlecht“ bis 10 „sehr gut“)	7,2 ± 3,2	6,0 ± 4,4	0,108
*Fatigue* (0 „gar nicht“ bis 10 „sehr oft“)	2,1 ± 3,2	4,4 ± 3,4	0,015*
*Schmerz* (0 „keine“ bis 10 „sehr starke“)	0,9 ± 1,7	2,9 ± 2,8	0,010*
*Dyspnoe*	–	–	0,220
Ja	7 (23)	23 (77)	–
Nein	9 (43)	12 (57)	–

Die Daten sind als Anzahl (%) für kategoriale Variablen und als Mittelwert ± Standardabweichung für kontinuierliche Variablen dargestellt

*n* Anzahl der Patienten

^1^ Kategoriale Variablen: X^2^-Test, Fisher-Exact-Test (zweiseitig), kontinuierliche Variablen: Mann-Whitney-U-Test

**Fett**: Signifikante Unterschiede, *p ≤ 0,05

Mithilfe der multivariablen logistischen Regressionsanalyse wurden Faktoren identifiziert, die unabhängig mit der „Wichtigkeit, körperlich aktiver zu werden“ assoziiert sind. Lebensgemeinschaft war signifikant mit dieser Variable assoziiert (*p* = 0,033), während die Symptome Dyspnoe (*p* = 0,056) und Schmerzen (*p* = 0,068) knapp die statistische Signifikanz verfehlten. Mit dem Modell können 21,7 % der Varianz der Zielgröße aufgeklärt werden (korrigiertes R^2^ = 0,217; *p* = 0,012) (Tab. 4).

**Tab. 4:** Ergebnisse der multivariablen logistischen Regression^1^ (*n* = 51)

Koeffizienten	B	SE	*p*-Wert	95 %-KI
(Konstant)	0,009	3,917	0,998	−7,890–7,907
Lebensgemeinschaft	2,040	0,926	0,033*	0,173–3,907
Schlafqualität	0,256	0,180	0,163	−0,107–0,619
Fatigue	0,166	0,146	0,260	−0,127–0,460
Schmerz	0,379	0,202	**0,068**^**†**^	−0,029–0,787
Dyspnoe	1,918	0,977	**0,056**^**†**^	−0,052–3,888
Alter	−0,035	0,049	0,482	−0,133–0,064
Zeit nach Erstdiagnose	0,006	0,008	0,501	−0,011–0,022

R^2^ = 0,327; angepasstes R^2^ = 0,217; F = 2,992; Signifikanz des Modells: *p* = 0,012*

*B* Regressionskoeffizient, *SE* Standardfehler, *95* *%-KI* 95 %-Konfidenzintervall für B

^1^ Abhängige Variable „Wichtigkeit, körperlich aktiver zu werden“

**Fett**: Signifikanz ^*^ ≤ 0,05; ^†^ ≤ 0,10

## Diskussion

Zentrales Ergebnis der Studie ist, dass Menschen mit HNC den Großteil des Tages mit sitzenden Aktivitäten verbringen und nur in einem geringen Umfang von etwa 3 h/Tag körperlich aktiv sind. Für die Mehrheit der Teilnehmer ist es jedoch wichtig, sich mehr zu bewegen. Für Alleinlebende und Teilnehmende mit Schmerzen oder Dyspnoe zeigen die Daten eine höhere Wichtigkeit dafür, aktiver zu werden.

Angesichts der begrenzten Erreichbarkeit von Menschen mit HNC für angeleitete und strukturierte Bewegungsprogramme [22, 29], waren wir überrascht, dass für zwei Drittel der Teilnehmer eine Steigerung ihrer körperlichen Aktivität wichtig ist. Diese Einstellung teilen HNC-Patienten mit einem hohen Anteil der gesunden Bevölkerung. Der Wunsch nach mehr körperlicher Aktivität wird jedoch häufig aufgrund vielfältiger Barrieren, wie z. B. geäußerter Zeitmangel oder mangelnde Motivation oder auch Rückfall in bewegungsarmes Verhalten in der kälteren Jahreszeit, häufig nicht in die Praxis umgesetzt [21]. In einer aktuellen Übersichtsarbeit bei vermeintlich Gesunden im Alter ab 55 Jahren erwies sich die Integration der drei Aktivierungsfaktoren „Motivation“, z. B. auf Basis eines personalisierten Feedbacks von Wearables, „Fähigkeiten“, z. B. durch die persönliche Interaktion mit dem Ziel, Bewegungsübungen selbstständig auszuführen, sowie „Wissen“, z. B. durch Aufklärung über die gesundheitlichen Folgen unzureichender körperlicher Aktivität, als effektiv dafür, eine Verhaltensänderung zu erzeugen. Interventionen, die allein die unbeaufsichtigte Nutzung digitaler Technologien beinhalten, wurden als nicht effektiv bewertet [3].

Aktuell wird in der Arbeitsgruppe der Erstautorin die Akzeptanz einer Smartwatch bei HNC-Patienten erprobt [33, 34]. Ihr Einsatz, in Kombination mit digital bzw. remote-gestützte Heimtrainingsprogrammen [13] oder interaktiven Webseiten, wie sie bereits zur Prävention von Schluckstörungen getestet wurden [30], ist in Planung.

Übereinstimmend mit anderen Studien [22, 29] zeigte sich bei der Erfassung der körperlichen Aktivität über sieben Tage, dass HNC-Patienten die meiste Zeit des Tages mit sitzenden Aktivitäten verbringen und ausschließlich mit leichter bis moderater Intensität aktiv sind. Besteht bereits ein höheres Aktivitätsniveau, ist es für die Teilnehmer auch weniger wichtig, noch aktiver zu werden. Hingegen ist es Teilnehmern mit einem niedrigeren Aktivitätsniveau wichtiger, sich mehr zu bewegen. Die hohen Sitzzeiten und deren bekannte negative Auswirkungen [9] sprechen für eine intensivere Aufklärung der Patienten, wonach „Erwachsene die Zeit, die sie sitzend verbringen, begrenzen sollten“ [35]. Die in der Stichprobe nahezu nicht vorhandenen intensiven Aktivitäten sprechen dafür, stärker als bislang Empfehlungen und betreute Bewegungsprogramme auch an Aktivitäten in leichter Intensität (z. B. Spazierengehen) auszurichten. Diese Schlussfolgerung wird durch Ergebnisse von Lønbro et al. [19] gestützt, wonach Alltagsaktivitäten bei Menschen mit HNC und geringer funktioneller Leistungsfähigkeit denselben positiven Effekt haben können wie Krafttraining.

Die Empfehlung bei HNC-Patienten körperliche Aktivitäten mit leichter Intensität zu erhöhen, resultiert ebenso aus dem Wissen, dass gesunde Erwachsene vergleichbaren Alters eine deutlich höhere Alltagsbewegung insbesondere im Bereich der leichten körperlichen Aktivitäten aufweisen [8, 36].

Alleinstehende Teilnehmer gaben häufiger an, dass es ihnen wichtig sei, körperlich aktiver zu werden, als Teilnehmer, die in einer Partnerschaft lebten. Überraschenderweise unterschieden sich die beiden Gruppen nicht hinsichtlich der erfassten körperlichen Aktivität. Möglicherweise spielt die Teilnahme am Gemeinschaftsleben für alleinstehende HNC-Patienten eine wichtigere Rolle als bei Anderen. Folglich sollten die Maßnahmen zur Bewegungsförderung in dieser Patientengruppe auch soziale Aspekte einbeziehen (z. B. Gruppentraining, Peer-Unterstützung, Selbsthilfegruppen).

Eine hohe Symptomlast, Komorbiditäten, Zeitmangel und mangelnde Motivation sind wesentliche Hindernisse für mehr körperliche Aktivität und schränken die Lebensqualität von HNC-Patienten ein [5, 24]. Das Ergebnis, dass Teilnehmer mit einer hohen Symptombelastung die Wichtigkeit von mehr körperlicher Aktivität betonen, diese einer Umsetzung von mehr Aktivität ggf. aber im Wege steht, unterstreicht die Notwendigkeit eines guten Symptommanagements [15].

Mit den von uns in die Regressionsanalyse einbezogenen soziodemografischen und klinischen Variablen kann zirka ein Fünftel der Varianz hinsichtlich der Wichtigkeit, körperlich aktiver zu werden, vorhergesagt werden. Dies ist vergleichbar mit den Ergebnissen von Buffart et al. [5], die bei 416 HNC-Überlebenden die Zusammenhänge zwischen demografischen, klinischen, lebensstilbezogenen und sozial-kognitiven Parametern, Absichten und Verhaltensweisen in Bezug auf körperliche Aktivität untersuchten. Die Autoren konnten mit ihrem Modell 22,9 % der Varianz in der Bewegungsintention erklären, wobei frühere Bewegungserfahrung, eine positive Einstellung zur Bewegung, soziale Unterstützung sowie ein hohes Maß an wahrgenommener Verhaltenskontrolle wesentliche Einflussfaktoren waren. Für das tatsächliche Bewegungsverhalten erklärten die untersuchten Faktoren 16,1 % der Varianz. Dieses war assoziiert mit jüngerem Alter, dem Fehlen unbeabsichtigter Gewichtsabnahme, dem Fehlen von Komorbiditäten, hoher Bewegungsintention sowie hoher wahrgenommener Kontrolle. Folglich spielen auch psychologische Variablen – insbesondere motivational-kognitive Aspekte – neben soziodemografischen und klinischen Merkmalen eine zentrale Rolle bei der Erklärung von Bewegungsverhalten in dieser Patientenkohorte.

Eine wesentliche Stärke dieser Studie ist die hohe Teilnahmerate. Die Analyse der soziodemografischen Daten zeigt, dass die Kohorte repräsentativ für Menschen mit HNC im in M‑V ist (Verhältnis Frauen zu Männern 1:4, niedriger Bildungsstand, Mehrheit Raucher oder ehemalige Raucher). Darüber hinaus gab es eine hohe Akzeptanz des Akzelerometers als objektives Maß für die Erfassung körperlicher Aktivität. Dennoch sind einige Einschränkungen dieser Studie zu berücksichtigen. Erstens rekrutierten wir auch HNC-Patienten in der lokalen Selbsthilfegruppe, die möglicherwiese motivierter hinsichtlich körperlicher Aktivität sind und eine höhere Studienaffinität aufweisen. Zweitens erfüllte fast ein Viertel der Teilnehmer nicht die Tragekriterien des Akzelerometers, was zu 19,6 % Datenverlust führte. Ob die Ausfälle systematisch waren, kann nicht beurteilt werden. Drittens ist die Stichprobengröße klein, sodass wir bei der Auswahl der statistischen Methoden eingeschränkt waren. Daher spiegeln *p*-Werte zwischen 0,05 und 0,1 nur schwache Belege gegen die Nullhypothese wider [17]. Viertens war der Beobachtungszeitraum für die körperliche Aktivität auf sieben Tage begrenzt. Fünftens wurde die Befragung kurz gehalten, um eine hohe Teilnahmequote zu sichern; daher wurde nur eine begrenzte Anzahl von Variablen, z. B. ausschließlich bewegungstherapierelevante Symptome, erfasst. Sechstens konnten die eingesetzten Beschleunigungsmesser aufgrund ihrer Technologie oder Tragebeschränkungen (z. B. bei Aktivitäten im Wasser) nicht alle Arten von körperlicher Aktivität erfassen (z. B. Ergometertraining).

## Ausblick

Empfehlungen zur körperlichen Aktivität in der onkologischen Versorgung sind bislang häufig sehr allgemein formuliert. Daten bzw. das Wissen um das Aktivitätsniveau in onkologischen Patientengruppen stellt eine wichtige Voraussetzung dar, um interventionell mit Maßnahmen zur Bewegungsmehrung anzuknüpfen. Hierbei sind Faktoren wie soziale Unterstützung und die Schwere insbesondere bewegungstherapierelevanter Symptome einzubeziehen. Dies trägt auch dazu bei, Ansätze zur Steigerung körperlicher Aktivität individueller zu gestalten – in Bezug auf Häufigkeit, Intensität, Umfang und Art. Es bedarf weiterer Forschung, um Empfehlungen und Anleitungen zur körperlichen Aktivität so auszugestalten, dass mehr als nur eine Minderheit der Patienten diese auch umsetzt.

## Fazit für die Praxis

- Zwischen der Motivation körperlich aktiv zu sein und der erfassten Aktivität besteht eine deutliche Diskrepanz – dies eröffnet Ansatzpunkte für Interventionen.
- Empfehlungen sollten nicht ausschließlich auf moderate oder intensive Aktivität fokussieren. Auch alltagsnahe, leichte Bewegungsformen können klinisch wirksam sein.
- Maßnahmen zur Bewegungsförderung sollten auch soziale Aspekte einbeziehen.
- Ein gutes Symptommanagement ist Grundlage jeder Bewegungsförderung.
- Bewegungsangebote sollten symptomorientiert individuell angepasst, möglichst niedrigschwellig, kostenfrei und flexibel gestaltet werden, um möglichst viele Patienten für eine Nutzung zu gewinnen.

## Data Availability

Die erhobenen Datensätze können auf begründete Anfrage hin, in anonymisierter Form bei der korrespondierenden Autorin Frau Dr. Sabine Felser, angefordert werden. Die für diese Studie generierten und analysierten Datensätze sind im NFDI4Health-Speicherort hinterlegt: http://ldh.mediz-rostock.imise.uni-leipzig.de/projects/15#datafiles.
